# The Pertussis resurgence: putting together the pieces of the puzzle

**DOI:** 10.1186/s40794-016-0043-8

**Published:** 2016-12-12

**Authors:** Rotem Lapidot, Christopher J. Gill

**Affiliations:** 1 Department of Pediatric Infectious Diseases, Boston Medical Center, Boston, MA USA; 2 Department of Global Health, Boston University School of Public Health, Boston, MA USA; 3 Center for Global Health and Development, Boston University School of Public Health, 801 Massachusetts Avenue, Boston, MA 02118 USA

**Keywords:** Pertussis, Acellular pertussis vaccine, Resurgence, Epidemiologic modeling, Asymptomatic transmission, Pertussis vaccines, Review

## Abstract

Pertussis incidence is rising in almost every country where acellular pertussis (aP) vaccines have been introduced, and is occurring across all age groups from infancy to adulthood. The key question is why? While several known factors such as waning of immunity, detection bias due to more sensitive tests and higher awareness of the disease among practitioners, and evolutionary shifts among *B. pertussis* all likely contribute, collectively, these do not adequately explain the existing epidemiologic data, suggesting that additional factors also contribute. Key amongst these is recent data indicating that the immune responses induced by aP vaccines differ fundamentally from those induced by the whole cell pertussis (wP) vaccines, and do not lead to mucosal immunity. If so, it appears likely that differences in how the two categories of vaccines work, may be pivotal to our overall understanding of the pertussis resurgence.

## Background

Ideally, vaccines should possess two key attributes: 1) Direct protection of the vaccinee by creating endogenous immunity rendering him/her resistant to the disease in question; and 2) Indirect protection of individuals that were not vaccinated, by preventing circulation of the pathogen. This is achieved by immune responses that also block acquisition, replication and transmission of the pathogen in or from the host, regardless of whether that infection actually led to clinical illness. The former protects individuals in a population from developing symptomatic disease, but that is distinct from whether infections per se are blocked. The latter hinders the pathogen’s movement through a population, and is the mechanism that leads to herd effect [1].

Perhaps the best example of this latter feature were the conjugated protein-polysaccharide vaccines targeting *Streptococcus pneumoniae*, *Haemophilus influenzae* type B, and *Neisseria meningitidis.* In each case, introduction of the vaccine led to a steep decline in clinical disease by vaccine serotypes/groups among those vaccinated, but also a significant decline among the non-vaccinated population, indicating herd effects. In each case, the critical mechanism underlying this herd effect was a reduction in nasopharyngeal carriage, which is itself mediated by mucosal immunity [2–7].

The resurgence of Whooping cough, caused by *Bordetella pertussis*, to many parts of the world, is a subject of great concern and debate. Part of this uncertainty rests in the limitations of our knowledge of pertussis disease pathogenesis and how natural or vaccine induced immunity impedes pathogen acquisition, replication, and movement through populations. Pertussis is classically described as a prolonged illness of paroxysmal coughs ending with an inspiratory whooping sound and post-tussive emesis. In the pre vaccine era the disease was responsible for hundreds of thousands of pertussis cases a year with severe or fatal cases concentrated among very young infants. From the 1950’s widespread immunization of children in the US with the whole cell (wP) pertussis vaccine led to a 99% reduction in pertussis cases, but not in complete elimination of the disease [8].

**Fig. 1:**
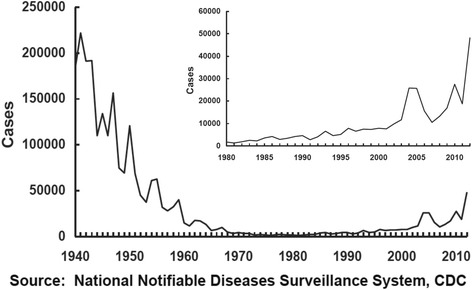
Pertussis cases in the US, 1940–2012. Data are from the Centers for Disease Control and Prevention via the National Notifiable Diseases Surveillance System

But while effective, the modest reactogenicity of wP vaccines and concerns about possible rare neurologic adverse events that might have been linked to wP vaccines prompted a need for a new, safer vaccine to replace it [9]. Following a lengthy development process, in 1997 the US switched to acellular vaccines (aP), which are far less reactogenic, and, based on early head to head clinical trials comparing the aP with the wP vaccines, appeared to be as protective as the wP. However, the wP vaccine chosen by the CDC to be used in those trials, manufactured by Connaught, was later found to be one of the most poorly immunogenic of available wP vaccines [10]. Several years later an increase in incidence of pertussis in children aged 7–10 years was seen, the first birth cohort to receive the aP vaccines [11]. By 2014 there were more than 32,000 cases reported in the US, the highest incidence since the 1950s (Fig. 1) [8, 12]. Other countries who also switched from wP to aP vaccines, including the UK, Australia, Canada, Spain, Belgium, and the Czech Republic had the same experience, with a rise in pertussis incidence after a 5–10 year lag from the wP to aP vaccine switch.

The key question, and the focus of this paper, is why? Since the resurgence of pertussis has occurred in almost every country where aP vaccines replaced wP vaccines, it cannot be assumed to be coincidence. Several explanations have been suggested including: waning of immunological response to the aP vaccine, detection bias, emergence of mutated *B. pertussis* strains not covered by the vaccine, and more. All are relevant theories, and each of them probably contributes to the reemergence of pertussis. However, none of these seem to adequately explain the pattern of rise of pertussis disease [12, 13], suggesting that a key piece of the puzzle is still missing.

As will be shown, the answer to this question likely rests on a multiplicity of sources of evidence, including epidemiologic studies, modeling exercises, experimentally derived data using animal models of pertussis exposure and vaccine responses, and recent insights into the immunology of pertussis and pertussis vaccines. A key theme that emerges is whether and to what degree aP vs. wP vaccines induce mucosal immunity, what the specific immunological mechanisms are that allow for that response, and how these differ between the two vaccine types.

## Analysis

### Characteristics of the wP vaccines

The introduction of the wP vaccines in the US in the 1950s yielded a 99% reduction in cases of clinical pertussis [8]. But while the vaccines clearly worked well, the specific immunological mechanisms that were essential to their effectiveness were never identified, nor the key antigens that induced these responses. A reminder of this ambiguity is the fact that we still do not have a clearly identified immunologic correlate of protection for wP vaccines, nor for aP vaccines. It should be noted that the wP vaccines, by their very nature, include 100 s or 1000s of non-standardized antigens at varying concentrations. Which of these, alone or in combination, are the essential ones remains enigmatic [14].

But even absent clear understanding of the immunology of wP vaccines, it is still possible to identify ways in which the wP vaccines appear to offer population level protection. The British epidemiologists Fine and Clarkson described the various methodological problems pertaining to the design and analysis of vaccine studies, that may contribute to variations in wP vaccine efficacy estimations [15]. Amongst others, they address perhaps the most important question of all: what do wP vaccines protect from? Do they mainly prevent disease? Or do they also prevent infection, irrespective of symptoms? Pertussis infection has a spectrum of presentations with the classical “whooping cough” on one end, through mild illness in re-infected and vaccinated individuals on the other end. Whether pertussis exists in a true ‘carrier state’ of asymptomatic infection has never been resolved, since no carriage studies have ever been conducted.

Studies that assess pertussis vaccine efficacy only against ‘severe’ disease would be predicted to show high efficacy, which emphasizes the direct effects of the vaccine. Naturally, if including milder or asymptomatic disease, then vaccines will show lower efficacy. As been shown by Fine and Clarkson with epidemiological data from England and Wales [15] milder cases of pertussis are probably harder to confirm, as they have a higher chance of being “culture negative”, and we assume perhaps also induce a milder serologic response as well. If that indeed is true- this could give a biased higher efficacy estimation. Most of the efficacy trials were confirming pertussis by culture and/or serologic response, and were actually focusing on the severe form and direct protection rather than milder or asymptomatic cases [15, 16].

### How the switch from wP to aP vaccines occurred

In 1986 the first placebo-controlled trial of an acellular vaccine was carried out in Sweden, supported by the NIH. Sweden was selected because at that time it was one of the few countries in Europe that did not administer wP pertussis vaccines routinely to infants, having been discontinued several years earlier in the wake of several highly publicized adverse events [17]. The trial was conducted in children aged 5–11 months old. 3801 children were given 2 doses of aP vaccine and were followed for 15 months after the second dose. The aP vaccine was found to give significant protection against laboratory confirmed pertussis (by culture or serology testing), the protection was better against more severe disease – a clear sign of robust direct effects of the vaccine – but the vaccine efficacy was not significant when cases that were not confirmed by laboratory tests were included [18]. Was this because these non-confirmed cases were in fact due to other pathogens than *B. pertussis*? Or, was this because the vaccine had limited effect in preventing mild disease and asymptomatic carriage (and therefor transmission)?

Another interesting fact about the environment in which the efficacy trials were held, is that at the time the clinical trial for aP were conducted, the population aged 7 years and older had already been vaccinated with wP vaccine, and presumably had some degree of protection against pertussis. In other words, the trial was not conducted in a vaccine-free environment that would allow to accurately assess the efficacy of the vaccine, but rather was influenced by the fact that there was some grade of protection in the majority of the population that could serve as the reservoir of pertussis.

A summary of the aP vaccine efficacy trials is shown in Table 1. The aP vaccines used in each trial contained different concentrations of antigens. All contained proteins thought to produce protective immunity: the pertussis toxin (PT), which is believed to be the major virulence factor, is found in all the vaccines. But they also contained other various antigens in different concentrations and combinations, such as filamentous hemagglutinin (FHA), Pertactin (PRN), and fimbriae (FIM) [19].

**Table 1:** Efficacy trials of acellular pertussis vaccine [10, 64, 65]

Study year	Study location/name	Design and methods	Number of participants	Comments.
1985 [18]	Sweden, Stockholm	Double blind placebo controlled (compared two Japanese aP vaccines)	3801	No wP control group. 2 dose schedule
1991 [66]	Sweden GÖteborg	Double blind placebo controlled (compared DT/DTP)	3450	No wP control 3 dose schedule.
1992 [67]	Germany, Mainz	Passive monitoring of household contacts	360 contacts	3 dose schedule
1992 [68]	Sweden, Stockholm	Double blind placebo controlled (two-compenent aP/five component aP/wP/DT)	24,336	wP control – (Conaught) 3 dose schedule
1992 [69]	Italy	Double blind placebo controlled (aP/wP/DT)	14,751	wP control – (Conaught) 3 dose schedule
1993 [70]	Germany, Munich	Case control study (aP/wP/DT/no vaccine)	16780	3 dose schedule
1990 [71]	Senegal	-Double blind placebo controlled -Household contact (aP/wP)	4181	No placebo control 3 dose schedule
Late 90′s [72]	Germany, Erlangen	Prospective study, 2 groups randomized to aP or wP, third group (not randomized) received DT.		4 dose schedule

The pivotal studies that ultimately led to the licensure and adoption of aP vaccines were conducted in Italy and Sweden, both selected because they had not incorporated pertussis vaccines into their infant schedules. In both, aP was compared to wP and against a placebo using a blinded, randomized controlled design, with culture or serology confirmed clinical pertussis as the primary endpoint. And in both cases, the aP vaccines proved to have far superior tolerability, improved safety, and yielding higher concentrations of antibodies against pertussis toxin compared with wP vaccines. In other words, a seemingly clear ‘win’ for the aP vaccines.

In light of the subsequent rise in pertussis following the switch to aP vaccines, it is worth considering how it was that these vaccines, initially so promising, ultimately proved so disappointing. The following features all likely contributed to favoring the efficacy of aP vaccines over that of wP.

First, when the different aP vaccines were compared to a wP vaccine and placebo, in the majority of the studies the whole cell vaccine was found to be more immunogenic. Only in two trials the aP vaccine had higher efficacy compared to the wP vaccine and those two trials were the pivotal trials leading the support to switch to the aP vaccine. Those were the trials in which the poorly immunogenic wP vaccine manufactured by Connaught, was used [10]. That, of course, would bias the study to show superiority of the aP vaccine.

Second, is that the immunogenicity of each vaccine was estimated by measurements of antibodies against PT and FHA, two of the antigens present in high concentrations in the aP vaccine. And indeed, far superior serologic responses, defined as a four-fold increase in antibodies titers, was seen to those antigens after immunization with the aP vaccines than the wP vaccines. Unfortunately, the implication of such response is poorly understood since we did not then, and do not now, have a clear correlate of protection for wP or aP vaccines. And since wP vaccines are a mixture of literally thousands of antigens, plus the lipopolysaccharide in the bacterial membrane, the precise mechanism underlying how wP vaccines worked was never elucidated. While PT and FHA are present, they are not present in the same concentrations in a wP vaccine as in an aP vaccine, and their relative importance to the overall protective effect of the vaccine is difficult to parse. Therefore showing that an aP vaccine produces higher anti-PT or anti-FHA antibodies than wP vaccines presupposes that these antigens are what drive immunologic protection, which was not shown to be true.

Third, the studies eliminated consideration of long term response to the vaccine, and there was limited evaluation of the vaccine overtime.

And lastly, the studies only measured the direct, not indirect, effects of each vaccine. As noted earlier, an important quality of a vaccine is its ability to induce herd immunity by preventing transmission of infection to un-immunized populations (i.e. newborns). The trials were not designed to evaluate that quality of the aP vaccines.

### Hypotheses for the resurgence of pertussis in the aP vaccine era

Over the years, a number of explanations for the resurgence have been postulated. These include: 1) Detection bias; 2) Poor persistence of antibodies; and 3) Evolutionary shifts among *B. pertussis* (waning or ‘leaky’ efficacy). Each will be discussed in turn, and while all may contribute, collectively they fail to fully explain the observed patterns of the pertussis resurgence.

1. Detection bias
*B. pertussis* is fastidious and intrinsically difficult to grow in culture. Moreover, cultures can be rendered ‘sterile’ by antibiotics, leading one to miss actual cases of pertussis. In the last 25 years the use of PCR has revolutionized pertussis detection, yielding results that are far more sensitive and specific than culture, and robust against the masking effects of antibiotic exposure. However, the down side to PCR’s sensitivity is that it detects a lot more pertussis, and in particular, identifies a higher burden of milder and atypical cases than did traditional culture methods [12, 20, 21]. It has been suggested that PCR may have revealed a hidden burden of disease, meaning that much of the apparent rise in cases is artifactual, and merely a reflection of improved detection methods [22–24]. While that argument has merit, and likely does explain the shift to a milder spectrum of illnesses being identified, it does not fully explain the observed data. In particular, increases in the rates of severe and fatal pertussis among infants have been recorded in the US and elsewhere after the switch to aP vaccines. In recent analyses, the WHO has determined that the rise in pertussis is real, and not artifactual [25].
2. Poor persistence
  The biological factors by which immunity to pertussis is achieved and maintained are complicated and poorly understood. Following infection and vaccination, antibodies against many *B. pertussis* antigens develop. It was postulated in the past that natural infection with *B. pertussis* caused close to lifelong immunity, but data regarding adult infection in unvaccinated populations indicate that immunity after natural infection wanes over time. Studies of unvaccinated children in the Netherlands and Senegal showed that reinfection was not uncommon and the duration of protection after first episode of pertussis lasted at least 7–10 years, but was not permanent [26]. However, this phenomenon appears to be far more pronounced with aP vaccines. This was first documented in a long term follow up of the Swedish children first to be vaccinated with the aP vaccine, whose susceptibility to pertussis appeared to have faded by 7–8 years of age [27]. More recent studies show that by 5 years after completion of a DTaP series, children were up to 15 times more likely to acquire pertussis compared to the first year after the series. Studies have also documented rapid decline in pertussis antibodies within as few as 2–3 years of the most recent aP vaccination, often to pre-vaccination levels [28–32] and although antibody levels alone are not necessarily indicative of waning immunity, in this case given the higher risk of infection after aP vaccine with time, it is strongly suggestive of it. When comparing the immunologic response to aP and wP, adolescents that were previously vaccinated with aP were at much higher risk (almost 6 times higher risk) to be infected with pertussis than those vaccinated with wP [33]. One study, examining US infants during the transition years from aP to wP vaccines, found that receipt of even one dose of wP vaccine in an otherwise purely aP vaccine series, led to significantly improved protection than infants who only received the aP vaccines [34].
  Thus, it is clear that lack of persistence of immunity after aP vaccine, and to some extent also after wP vaccine and natural infection, plays an important role in maintaining pertussis within populations. But logically, if the problem is merely poor persistence, one would anticipate that increases would occur first among adolescents, and latest among younger children. However, the pertussis rise was synchronous across all age groups, meaning that disease transmission was occurring across all age groups in parallel [13, 35]. So while an important factor in the pertussis resurgence, other data suggest that this is only one piece of the puzzle.
3. Evolutionary shifts among *B. pertussis* (waning or ‘leaky’ efficacy)
  Just as exposure to antibiotics creates a selective evolutionary pressure for bacteria to develop resistance to antibiotics, so too can vaccines exert pressure for bacteria to evolve to different antigenic isoforms of proteins included in vaccines [36–41]. In the case of *B. pertussis*, this adaptation has been clearly demonstrated to occur via at least three known mechanisms – antigenic shift away from antigens covered by the aP vaccine, deletion of antigens covered by the aP vaccine, and over-production of antigenic targets. In a comprehensive study spanning over the past 100 years, Bart et al. [42] analyzed genome of 343 disease causing *B. pertussis* isolates collected from 19 countries around the world. The major changes in antigen gene alleles were from ptxA2 to ptxA1, fim2-1 to fim2-2 and ptxP1 to ptxP3: in each case a shift from the specific antigen alleles included in aP vaccines to alleles that are not covered by the vaccine. Mooi et al. [36] found in the late 1980’s that *B. pertussis* strains emerged with increased production of pertussis toxin. In this case, the PT promotor showed a relatively high degree of polymorphism, suggesting the PT over-production has adaptive value. These new strains, carrying the ptxP3 allele, were more virulent to humans with higher incidence of hospitalizations and deaths, and this appears to have reflect that the ptxP3 promoter far over-expresses pertussis toxin compared with the ptxP1 promoter [36, 37, 43–46]. An even more definitive evolutionary escape route has been noted from multiple laboratories regarding pertussis isolates that stopped expressing one or more of the aP antigen genes entirely. This includes, in isolation or combinations, Pertactin, FHA and more recently PT. [45, 47–49] Remarkably, PRN expressing *B. pertussis* strains have entirely disappeared from the US, and the finding of PT-minus strains is surprising given assumptions that pertussis toxin was an obligatory virulence factor.
  But as with the issue of poor persistence, evolutionary shifts to evade aP vaccines do not appear to satisfactorily explain the pertussis resurgence. One problem with this explanation is Sweden, where a pertussis toxin-only, mono-valent aP vaccine has retained efficacy for over 20 years, despite the accumulation of non-aP vaccine allele strains of pertussis. Another problem is the timing of these shifts, which accumulate gradually with time. By contrast, the increase in pertussis disease in countries that switched to aP vaccines has, in each case, occurred after a now stereotypical lag of 5–10 years, not at all the pattern that one would expect if the problem was due to the slow acquisition of mutations. Moreover, the degree to which escape mutants of pertussis contribute to the overall resurgence has not been established.
  Thus as with detection bias and poor persistence, evolution may be a contributing factor to pertussis resurgence, but something is still missing.

### Recent insights from mathematical models of pertussis transmission

Probably one of the most significant finding from recent years is that aP vaccines provide strong direct protection against severe disease (at least in the short term), but may have relatively little indirect effect on transmission.

The lack of protection from transmission of the pertussis vaccines was first hypothesized by Fine and Clarkson in 1982 [50]. Fine and Clarkson contrasted the inter-epidemic cycle length during a period of high wP uptake in the UK against a subsequent period of low wP uptake in the UK (due to concerns from serious adverse events). Mathematical modules of epidemiology of pertussis consistently show that the disease peaks every 3–4 years. Fine and Clarkson found no change in the cycle length and therefore concluded that wP did not impede transmission, i.e., that wP lacked herd effect.

This conclusion was influential but appears to have been flawed for several reasons. First and foremost, Fine and Clarkson looked at the epidemic cycle lengths over time using national case averages from the UK. But that assumes that pertussis transmission is occurring synchronously, which is actually unlikely to be the case. Failure to account for local transmission epidemics in aggregate data would tend to blunt the shapes of epidemic cycles, and could obscure true changes in the cycle length. To account for this, in 2000 Rohani et al. [51] compared the inter-epidemic cycles before and after the introduction of wP pertussis vaccine in different cities in the UK, using each city as its own comparator. In each case they found that the interepidemic cycle was increased by 1.5-2.5 years by the introduction of wP vaccines. This observation has now been replicated in 64 countries around the world [52].

Following this same reasoning, but taking the switch instead from wP to aP vaccines as the reverse test case, Althouse and Scarpino, using a technique called ‘wavelet analysis’ (essentially a way of looking at multiple small outbreaks of disease over time), found a re-contraction of the interepidemic cycle length after the wP to aP switch. That is precisely what would be expected if wP vaccine block transmission and disease, but aP vaccines only block symptomatic disease, and have little impact on transmissions [13].

In a parallel analysis, Althouse and Scarpino examined the pace of genetic mutation among observed cases. Their surprising finding was that genetic mutation seemed to outpace known routes of transmission, suggesting that the extra genetic diversity reflected longer transmission pathways between observed cases, and hence the existence of asymptomatic carriage and transmission. This was a seminal observation, and we will return to this issue subsequently.

Indirectly, these models also shed light on the surprising failure of Cocooning to protect young infants from pertussis. Cocooning refers to the practice of administering Tdap to all household contacts of a newborn, as a means of providing an immunologically safe cocoon, shielding the newborn from infectious contacts. However, the success of Cocooning presupposes that aP vaccines block carriage and asymptomatic transmission in addition to blocking disease. This practice proved surprisingly unsuccessful in randomized controlled trials. This seems counterintuitive if we assume that aP vaccines block transmission and disease, but are exactly what would be expected if they permit silent infections and continued transmission [53, 54].

In summary, the mathematical models suggest that wP induced protection against transmission to a significant extent, meaning that they blocked transmission, offered herd immunity, and likely did so by blocking carriage, whereas the aP vaccine had no or minimal impact on blocking infections or transmissions, afforded poor herd effect, and, we would predict, do not block carriage.

### Differential impact of aP and wP vaccines in a baboon model

Probably the strongest evidence that aP vaccine fail to protect from transmission came from a series of studies by Warfel and Merkel at the US FDA using a non-human primate model involving infant baboons [55–58].

**Fig. 2:**
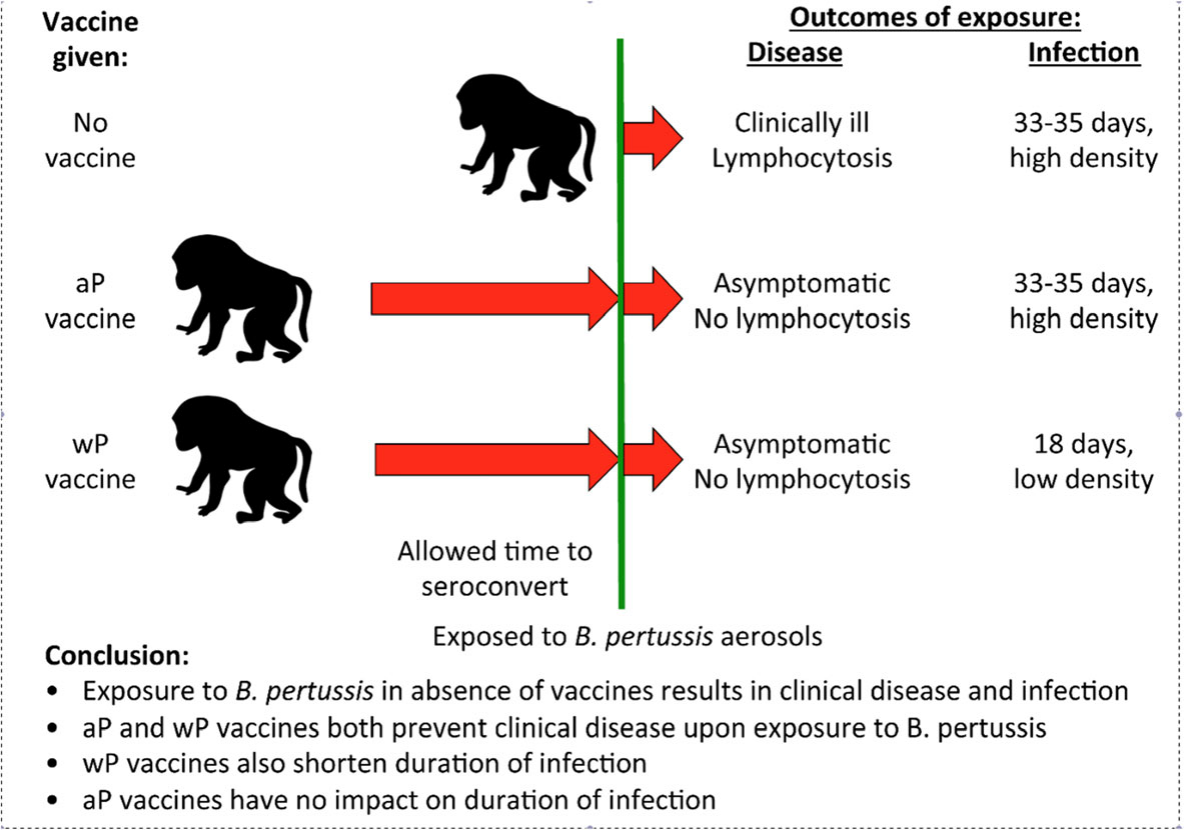
Outcomes of experimental exposure to pertussis among aP or wP vaccinated or unvaccinated infant baboons. Three groups of infant baboons were exposed to infectious aerosols of *B. pertussis*: unvaccinated control animals; animals vaccinated using acellular pertussis vaccines; animals vaccinated using whole cell pertussis vaccines. The vaccinated animals completed a full vaccination series and were allowed time to fully seroconvert before exposure. The two vaccinated groups of animals both remained asymptomatic, whereas the unvaccinated animals developed clinical disease. However, nasopharyngeal sampling of the three groups showed that the aP and unvaccinated animals both were infected with similar densities of pertussis bacteria and for similar durations. By contrast, the wP vaccinated animals were more resistant to carriage, and carried bacteria for shorter periods. This showed that wP and aP vaccinations induce very different mucosal immune responses, with the former protecting against infection and disease, and the latter only preventing clinical disease, but not infection

With Warfel’s Baboon model, we are now able to better understand the pathogenesis and the immune response to the disease and to the different vaccines. Baboons that were vaccinated with aP or wP vaccines were later challenged with aerosols of *B.pertussis*. Both sets of vaccinated baboons remained clinically asymptomatic, while unvaccinated control baboons became severely ill, and developed profound lymphocytosis, a common finding among human infant pertussis disease, and known to be mediated by pertussis toxin specifically (Fig. 2). This confirmed that aP and wP vaccines both provide excellent direct protection against disease (at least in the short term), and that pertussis disease in the animal model was a valid proxy for disease in human infants.

But when looking beyond disease to the vaccines’ efficacy vs. infection, the aP and wP vaccines diverged sharply. The un-vaccinated and aP vaccinated baboons remained colonized for an average of 33–35 days before clearing. By contrast, the wP vaccinated baboons cleared carriage after only 18 days, and had lower bacterial concentrations than the aP and vaccine-naïve animals.

**Fig. 3:**
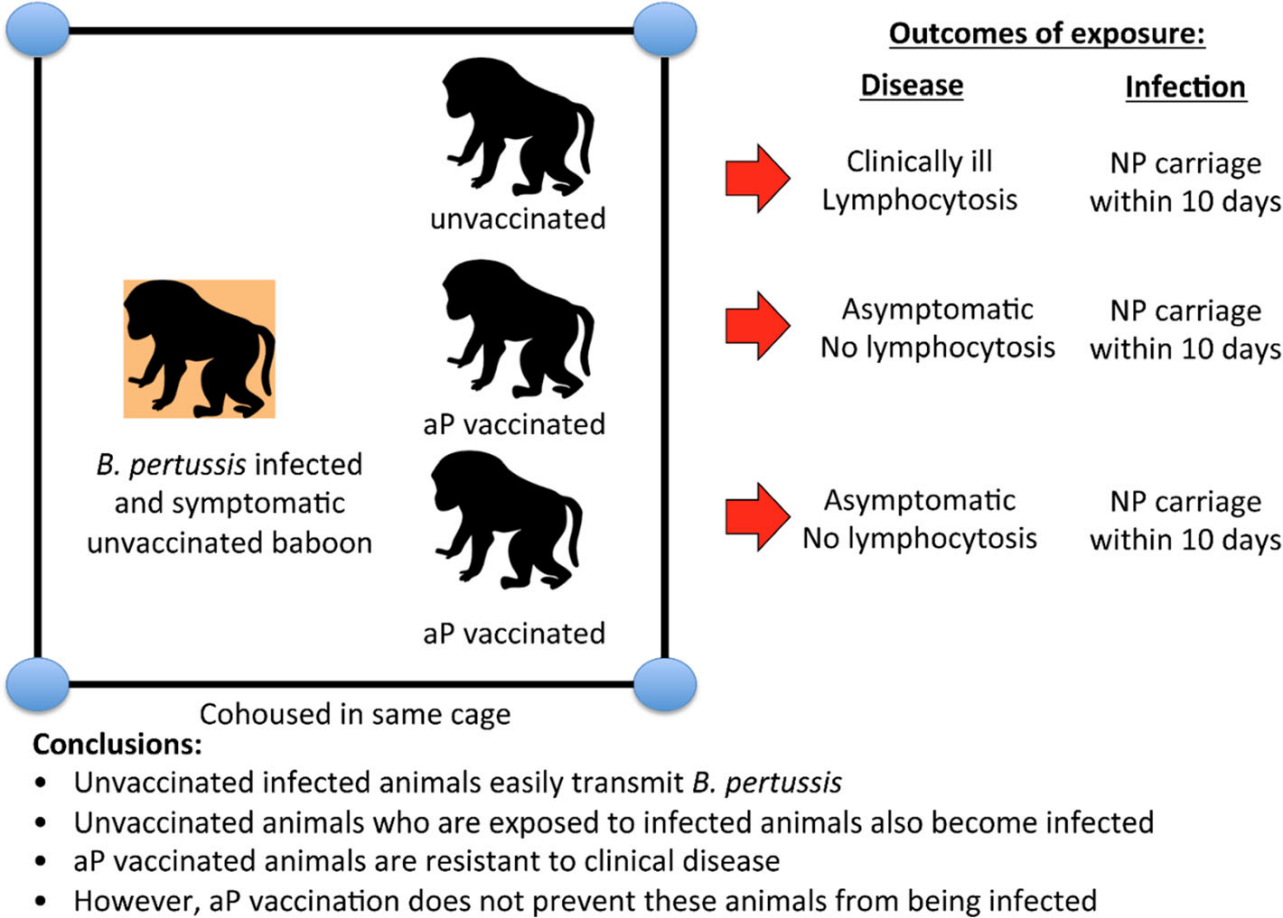
Outcome of exposure to infected animal by vaccination status. Here, an infected unvaccinated animal was co-housed with three initially uninfected animals, one of which was unvaccinated, while the other two had received aP vaccinations. All three animals became infected based on nasopharyngeal sampling, though only the unvaccinated animal showed signs of clinical illness. This showed that infection due to exposure to an infected animal can transmit *B. pertussis* (a more realistic model than exposure to aerosols). But again, while aP vaccinations blocked clinical disease, they did not prevent infection

**Fig. 4:**
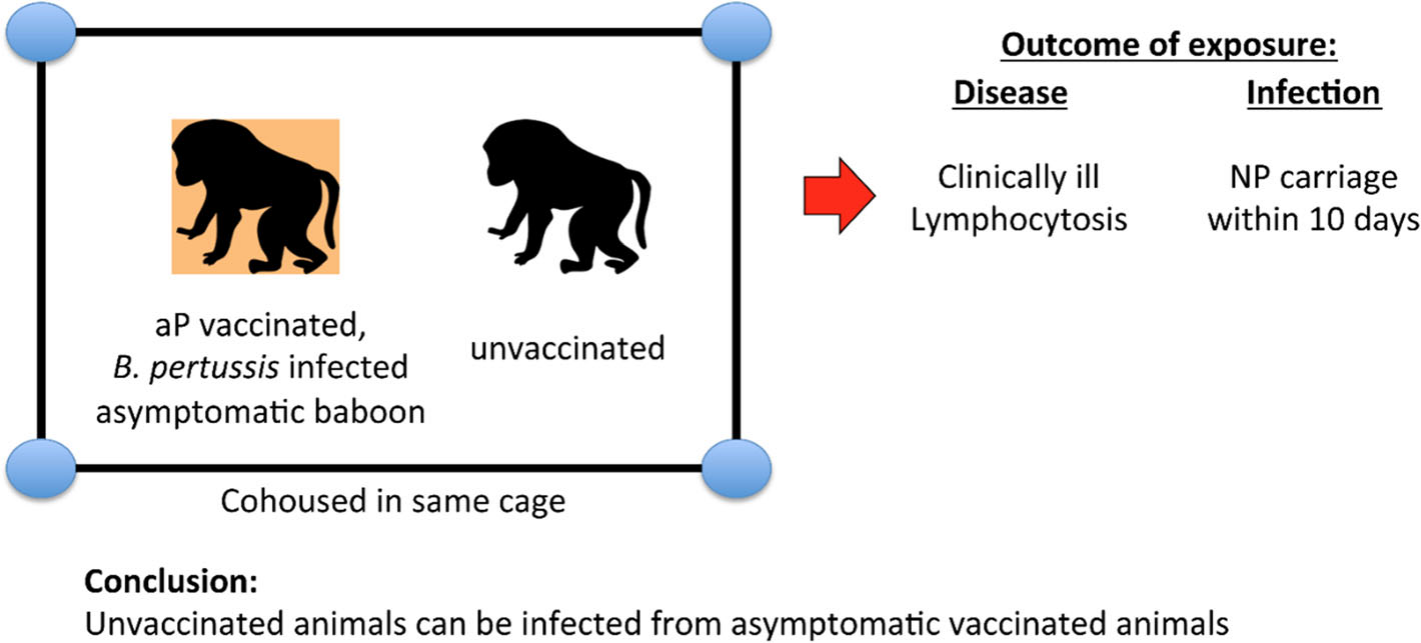
Infections from an asymptomatic vaccinated to unvaccinated animal. The first experiment showed that an unvaccinated but infected animal is capable of infecting an aP vaccinated animal. This experiment approaches this dynamic in reverse: here an aP vaccinated animal was infected with *B. pertussis* and then co-housed with an unvaccinated animal. Despite being asymptomatic, the aP vaccinated animal quickly infected the vaccine naïve control animal, who developed clinical disease as well as nasopharyngeal carriage showing that infection had occurred. This proved that, despite being symptom free, aP vaccinated animals can become infected with pertussis, and are able to transmit to unvaccinated animals. In other words, aP vaccination only prevented clinical disease, but did not prevent animals from being infectious and contributing to chains of transmission

Taking this one step further, when two aP vaccinated baboons and one unvaccinated baboon were co-housed in the same cage with an unvaccinated and infected animal, all three animals were colonized with pertussis within 10 days, further evidence that aP did not protect against colonization (Fig. 3). Taking this in reverse, when an aP vaccinated baboon was infected with *B. pertussis*, and then co-housed with a naïve baboon, the naïve baboon quickly became infected and became symptomatic (Fig. 4). Collectively, these experiments showed that both wP and aP vaccines prevent clinical disease, but that aP vaccines do not block infections. Moreover, aP vaccinated, asymptomatic but infected animals are quite capable of infecting other animals, and thereby participating in chains of transmission.

### Immunological insights into pertussis infections and vaccine responses

When looking in to the Immunological processes involved in conferring mucosal sterility, a recent discovery sheds more light and adds another missing piece to our puzzle. Until recently, the two classical known pathways for T helper cells were believed to be either the Th1 or the Th2 pathways. The former response primes T cells to control intracellular infections, such as tuberculosis; while the latter, Th2, optimizes anti-body responses against extracellular targets, and is involved in responses to parasitic infections. However, this dichotomy feels incomplete, since it fails to accounts for many T-cell mediated immune responses that do not neatly fit into either paradigm, many of which became clear as a consequence of the HIV/AIDS pandemic.

More recently, a third pathway was discovered of an independent lineage of cells, called the Th17 cells (so named due to dominant cytokine that drives cells down this pathway, interleukin 17). Of relevance, Th17 cells are found in the lungs, the gut mucosa, the skin, and play a critical role in mucosal immunity and host defense against extracellular pathogens [59, 60].

Following this logic, Warfel et al. also showed that infection with *B. pertussis* induced a pure Th17 response, and these cells were found to play a distinct role in the pathogenesis of *B. pertussis* infection [61]. Similarly, wP vaccines also induce a Th17 dominant responses (with a lesser Th1 contribution). By contrast aP vaccines only produced a Th2 response [56, 58, 62, 63].

## Conclusions

Table 2 summarizes the major differences between wP vaccine and aP vaccine as the evidence of the last 75 years suggest.

**Table 2:** Summarization of the major differences between wP vaccine and aP vaccine

Whole cell pertussis vaccine (wP)	Acellular pertussis vaccine (aP)
-Blocked disease and infection to a much higher extent than aP -Probably did this by blocking carriage (though studies were never done to prove that hypothesis).	-Blocked disease but did not block infection. -Experimentally do not block carriage and permit transmission from asymptomatic to vaccine naïve animals. This observation closely mimics the cocooning strategy, and suggests a reason why it had failed to perform as anticipated.
-Epidemiologically appears to offer herd effect (though not to a level sufficient to halt transmission entirely).	-Epidemiologically offers minimal or no herd effect.
-Induces robust mucosal immunity.	-Seemingly absent of mucosal immunity induction.
-Induces robust Th17 responses.	-Induces Th2 response.

The resurgence of pertussis likely has many contributing factors. And while detection bias, poor persistence, and leaky vaccine efficacy due to evolutionary shifts likely contribute to varying degrees in the pertussis resurgence, it seems far more likely that the key factor is instead immunologic. As with the conjugated protein-polysaccharide vaccines, the overall effectiveness of a pertussis vaccine when used at scale in a population is a function of direct and indirect effects. The lack of sterilizing mucosal immunity following aP vaccinations appears to be a critical limitation to these vaccine’s overall effectiveness, and in our view may be the most important factor of all in accounting for the resurgence.

If so, the implications of this inference are quite profound. The resurgence of pertussis in the past 2 decades is at once a public health and a public relations crisis. Vaccine hesitancy rates are rising, and the population is increasingly skeptical about professional pronouncements regarding vaccine policy. With the introduction and expanded use of aP vaccines into the population failing to control the rise in pertussis incidence, it seems increasingly likely that radical solutions will be required. This may include the resumption of wP vaccinations in some part of the infant schedule, or even the development of an entirely new pertussis vaccine. While it is too soon to know how this will play out, understanding how any new or improved pertussis vaccine affects mucosal immunity will be essential.
